# Bone Loss Rate May Interact with Other Risk Factors for Fractures among Elderly Women: A 15-Year Population-Based Study

**DOI:** 10.4061/2010/736391

**Published:** 2010-02-22

**Authors:** Joonas Sirola, Anna-Kaisa Koistinen, Kari Salovaara, Toni Rikkonen, Marjo Tuppurainen, Jukka S. Jurvelin, Risto Honkanen, Esko Alhava, Heikki Kröger

**Affiliations:** ^1^Bone and Cartilage Research Unit (BCRU), University of Kuopio, 70211 Kuopio, Finland; ^2^Department of Orthopedics and Traumatology, Kuopio University Hospital, 70211 Kuopio, Finland; ^3^Department of Obstetrics and Gynaecology, Kuopio University Hospital, 70211 Kuopio, Finland; ^4^Department of Clinical Physiology & Nuclear Medicine, Kuopio University Hospital, 70211 Kuopio, Finland; ^5^Department of Surgery, Kuopio University Hospital, 70211 Kuopio, Finland

## Abstract

Aim was to investigate fracture risk (FR) according to bone loss (BL) rate. A random sample of 1652 women aged 53.5 years was measured with dual X-ray absorptiometry in femoral neck in 1989 and 1994 and divided into tertiles of annual BL rate: high >0.84%, moderate 0.13%–0.84%, and low <0.13%. Low trauma energy fractures during following 10 years were recorded. There were no differences in FR between BL tertiles in Cox regression model. Factors predicting lower FR in Cox model were in high tertile: high T-score (HR 0.71; 95% CI 0.54–0.93, *P* = .012), no sister's fracture (HR 0.35; 0.19–0.64, *P* = .001), no mother's fracture (HR 0.52; 0.31–0.88, *P* = .015), in moderate tertile: high T-score (HR 0.69;0.53–0.91, *P* = .008) and good grip strength (HR 0.98; 0.97–0.99, *P* = .022). In low tertile there were no predictors for FR. BL predicted FR in women with mother's fracture in univariate and multivariate model (OR 2.6; 1.15–5.7, *P* = .021) but with sister's fracture this was observed only in multivariate model (OR 2.66; 1.09–6.7, *P* = .039). Accordingly, the risk factors for postmenopausal fractures, especially mother's fracture, may interact with BL.

## 1. Introduction

Osteoporotic fractures result in significant morbidity and mortality [1, 2]. The risk of fractures is greater among women with low BMD although it has now been accepted that differences in BMD and its changes explain only part of the increased fracture tendency among the elderly [3]. Other parameters of bone quality may be more important measures of fragility [4, 5]. Overall, the individual fracture risk is a sum of falling propensity and bone material quality and accordingly may be modified by genetic predisposition and several external environmental risk factors. 

 The diagnosis of osteoporosis is currently based on central DXA. Moreover, it has been emphasized that several non-BMD dependent risk factors should be taken into account while considering individual risk of fractures [6]. Accordingly, the recent trends in both clinical as well as research fields have favored a holistic and individually tailored approach to increased bone fragility instead of concentrating only on fractures related to osteoporotic BMD. As an example, a tool for the identification of 10-year fracture risk, FRAX, has been recently introduced for clinical use, which combines independent risk factors for fractures with BMD [6]. 

 Several studies have investigated the risk factors for osteoporotic fractures [7–13]. According to these studies, the most important environmental risk factors for low-trauma energy postmenopausal fractures are low BMD, previous fracture history, body composition, menopausal transition, excessive alcohol consumption, and smoking [7–12]. In addition, a family history of fractures has been suggested to be associated with increased postmenopausal risk of bone fragility in number of epidemiological studies [13–18]. 

 Previous studies have shown that the most cases of fragility fractures occur not among osteoporotic but among osteopenic women [19]. These findings reveal that there is more to postmenopausal bone fragility than BMD alone. However, no long-term population-based study has so far directly explored the differences in risk factors for fragility fractures according to rate of bone loss. The rationale for such a study would be the identification of risk factors for “high bone loss fragility disease” versus “other types of bone fragility disease”. In addition, the clinical tools for fragility fractures use cross-sectional or past information for evaluation of long-term fracture risk, which raises the question of whether changes in BMD or other risk factors in due course would significantly affect the estimated fracture risk. Accordingly, epidemiological data on effects of BMD change rate in conjunction with other risk factors for fractures is needed. 

 The aim of the present study was to identify the risk factors for perimenopausal low-trauma energy fractures according to bone loss rate. The primary hypothesis was that risk factors that account for low-trauma energy fractures in women with high and low bone loss rates are different.

## 2. Materials and Methods

### 2.1. Study Population and Study Frame

The study population was formed based on the prospective Kuopio Osteoporosis Risk Factor and Prevention (OSTPRE) study cohort. The OSTPRE cohort was established in 1989 by selecting all women born in 1932–1941 and resident in Kuopio Province, Finland (*n* = 14  220) [20]. The baseline postal inquiry, including questions, for example, about health disorders, medication, use of hormone therapy (HT), gynaecological history, nutritional habits, calcium intake, physical activity, alcohol consumption, smoking habits, and anthropometric information, was sent to these women at baseline in 1989. The five-year (in 1994), ten-year (1999), and 15-year (2004) followup questionnaires were sent to the 13 100 women who responded at baseline. Of these women, 11 954 responded to postal inquiry in 5-year, 11 537 in 10-year, and 10 926 in 15-year followup. Albeit the study design, and selection of the random sample, was prospective, the present study was performed by stratifying the study group of interest, described below in detail, retrospectively. The postal inquiries included informed consent from the participants and the study protocol has been accepted in the ethics committee of University of Kuopio and Kuopio University Hospital. 

**Figure 1:**
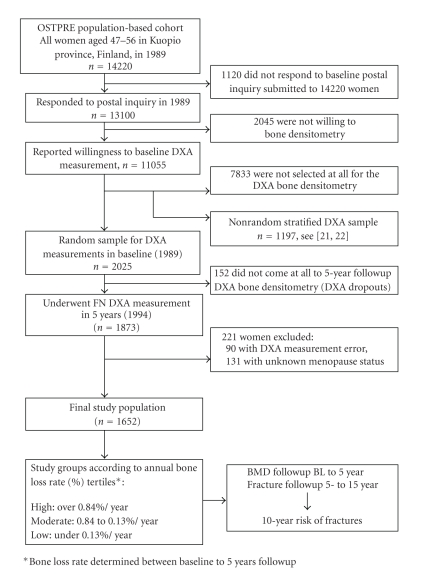
Study groups and study protocol (*n* = 1652).

 The selection of the present study population and study protocol is outlined in Figure 1. Of the 13 100 respondents to baseline postal inquiry in 1989, 11 055 (84.4%) reported willingness to undergo DXA densitometry. A densitometry sample of 3686 women (33.3%) was invited to the baseline DXA measurements while rest of the 11 055 women were not measured at all. Out of the women invited to DXA, 3222 (87.4%) women actually underwent the baseline measurement (i.e., 464 of the invited women did not participate at all). The women that actually underwent the baseline DXA were divided into two groups: random population-based sample consisted of 2025 women and the remaining 1197 women formed a nonrandom part which was stratified for other study purposes or were labeled to have a high-risk profile (i.e., experienced menopause within 2 years, had certain diseases/medications affecting bone, had multiple behavioral risk factors, were selected for an HT + vitamin D trial, or were included in additional rheumatoid arthritis sample [21, 22]). Accordingly, the non-random part (*n* = 1197) was *excluded *from the present study. In all, 1873 women of the random part underwent 5-year follow-up bone density DXA measurement (i.e., DXA dropout *n* = 152). Serial valid measurements for neck of femur were recorded for 1783 women in both baseline and the 5-year follow-up measurement. Accordingly, severe bone deformities, including deforming arthritis, and prostheses among other inaccuracies, (*n* = 90) were excluded by a systematic manual review of densitometry reprints by the study-group physicians.

Hysterectomized women and premenopausally bilaterally ovariectomized women were included in the study sample but women with unclear transition to postmenopause (either surgical or natural) were excluded (*n* = 131). The beginning of menopause was defined in this study as 12 months' amenorrhea [23]. The beginning of amenorrhea was based on self-reports about the last natural periods in the inquiries and no hormonal samples were collected in this respect. Accordingly, the final study population consisted of a random sample of 1652 perimenopausal women aged 53.5 (SD 2.9) years at the beginning of the study (year 1989) (Figure 1). 

 The present study population was divided into tertiles according to bone loss rate based on annual change in BMD (%) between baseline and 5-year follow-up measurements (Figure 1). The bone loss rate during the first 5-year follow-up was used for prediction of the following 10-year (5- to 15-year) fracture risk and an other 5-year measurement and postal inquiry risk-factor information were used as additional predictive data.

### 2.2. Fractures

Fractures during the 15-year follow-up period (1989–2005) were recorded based on questions (in baseline, 5-year, 10-year, and 15-year follow-up postal questionnaires) on whether the respondent had suffered a low-trauma energy fracture during the follow-up and, if so, the type, mechanism, circumstances, and treatment of the fracture. All self-reported fractures were validated by cross-checking radiological reports from medical records by study-group physicians. However, rib fractures were accepted without radiological evidence if the clinical diagnosis in the medical records was clearly and uniformly a rib fracture. For the present study, only low-trauma energy fractures were accepted, that is, stumbling, tripping, slipping, or falling under 1-meter height. The false positive rate in self-reported fractures was 16.5% and the false negative rate was 21.6%, respectively [24]. Because the prospective fracture follow-up of the present study was from 5- to 15-year follow-up, the fractures during the first follow-up interval (i.e., baseline to 5 years) were considered past fractures in multivariate models. 

 History of fractures of the first-degree relatives was inquired by the following questions: “have your *mother * suffered a fracture of wrist or hip or both?” and “Have your *sister* suffered a fracture of wrist or hip or both?”. Sister's and mother's fractures were treated as separate dichotomous (fracture/no fracture) variables in the analyses and all fractures either before or during the 15-year follow-up were taken into account. Accordingly, the either relatives fracture was considered from the view of genetics rather than an “a priori” risk factor. Also, the mother's and sister's wrist and hip fracture history was not questioned earlier than 10-year follow-up postal inquiry. The exact types/classification of relatives' fractures or trauma energy were not more specifically questioned and accordingly we were not able to consider this issue in the present study in more details. In statistical analyses women without confirmation of having a sister were considered missing values. The fractures of the father and brother(s) were also inquired with separate question, but not included in the analyses of the present study, because of insufficient number of fractures of these specific relatives for statistical purposes.

### 2.3. Other Variables of Interest

The height and weight of each study subject were measured with calibrated scale in the presence of study-group nurses at the time of each bone densitometry. The body mass index (BMI) was calculated as weight/height^2^ (kg/m^2^) in each follow-up measurement. 

 The use of hormone therapy (HT) was calculated based on the self-reported use of oestrogen containing tablets and patches and used for menopausal symptoms, which was specifically questioned in the inquiries. Three groups were formed according to menopause status and the use of HT during the menopausal transition: (1) *premenopausal* group of women who were premenopausal at 5-year measurement (year 1994) and underwent the menopausal transition during the 5- to 15-year follow-up but had not used HT, (2) *postmenopausal* group of women who were postmenopausal in 5-year measurement but had not used HT, and (3) *HT users* that included all women reporting use of HT for menopausal symptoms. This three category menopause variable was used as covariate in statistical models. The validation of self-reported use of HT with national medical prescription records has shown good correlation (98% of HT users were true users) and has been described in detail previously [25]. 

 Grip strength, measured with pneumatic hand-held dynamometer (Martin Vigorimeter, Germany), was taken to be the mean of three successive measurements. The measurements were done in controlled sitting position by trained nurses. Grip strength change was determined by change in age-adjusted grip strength quartile between baseline and 5-year measurements, because the measurement device was changed during the follow-up period. The intraclass correlation coefficient (ICC) of grip strength measurements has been shown to be 0.87 and for grip strength change measurements 0.85 [26]. The grip strength measurement protocol, and the validation of use of age-adjusted quartiles for grip strength change estimation, of the present study has been described in more detail previously [26]. 

 The alcohol intake (g/week) was calculated based on the following question: “how many drinks of beer/wine/spirits do you consume monthly on average?” in postal inquiries. The information on smoking was based on following questions: “have you ever smoked (cigarettes, pipe, etc.)?” and “do you smoke currently?” It was used as a dichotomous covariate in statistical models: (1) no smoking and (2) any smoking. The calcium intake of each participant was calculated according to self-reported ingestion of milk products in postal inquiries. The following questions were asked: “how many decilitres of fluid milk products (milk, sour milk, yoghurt, etc.) do you consume daily? ”, and “how many slices of cheese do you eat daily?” The amount of calcium was approximated to be 120 mg/dl for fluid milk products and 87 mg/slice for cheese.

### 2.4. Bone Mass Measurements

The DXA measurements of anterior-posterior spine (L2-L4) and femoral neck were carried out using the same Lunar DPX scanner in both baseline and 5-year measurements with the imaging and analysis protocols provided by the manufacturer (Lunar Co. Madison, WI, USA) and described earlier [21]. The measurements were carried out in Kuopio University Hospital by specially trained nurses. Quality standards were tested on daily basis. The short-term reproducibility of this method has been shown to be 0.9% for lumbar spine and 1.5% for femoral neck BMD measurements [27]. The long-term reproducibility (CV) of the DXA instrument for BMD during the study period, as determined by regular phantom measurements, was 0.4% [22]. For the purposes of the present study, BMD was converted into T-score according to the Finnish reference values, also adopted for clinical use, and described earlier [27].

### 2.5. Statistical Methods

Statistical analyses were carried out using the Statistical Package for Social Sciences (SPSS ver. 15, SPSS Inc., Chicago, Illinois, USA) for Windows. Kaplan-Meier curves were obtained to evaluate the fracture-free survival rate and Cox proportional hazard model to receive the corresponding hazard ratios (HR) and statistical differences (*P*-value). The mean annual bone loss rate (% of baseline BMD) was calculated according to following formulas:

(1) $$\begin{array}{cc} & \text{annual}\;\;\text{BMD}\;\;\text{Change}\;\;\left(\text{ABC}\right)\\ & \;\;=\frac{\left[\text{BMD}\;\;\text{at}\;5\text{-year}\;\;\text{follow-up}-\text{BMD}\;\;\text{at}\;\;\text{baseline}\right]}{\text{follow-up}\;\;\text{time}},\\ & \text{annual}\;\;\text{bone}\;\;\text{loss}\;\;\text{rate}\;\;\left(\text{\%}\;\;\text{of}\;\;\text{baseline}\;\;\text{BMD}\right)\\ & \;\;=\left[\frac{\text{ABC}}{\text{BMD}\;\;\text{at}\;\;\text{baseline}}\right]\times 100. \end{array}$$

The multivariate variables included (with references to publications including detailed information on building the covariate variables, if available, in brackets) are age, baseline BMI, fracture history before the baseline (yes/no), menopause status and use of HT (yes/no), grip strength [26], alcohol intake (mg/day), smoking (yes/no) [28], nutritional calcium intake (mg/day) [29], mother's fracture (yes/no), sister's fracture (yes/no), and morbidities and medication potentially affecting bone (yes/no). The categorical variables were entered into the Cox proportional hazards model as indicator variables. Interaction analysis was performed by entering the term [bone loss × variable] in to the Cox model simultaneously with both independent variables. The selection of conditions potentially affecting bone (used in covariate models) has been described previously by Kröger et al. [21]. One combined dichotomous variable (any bone-affecting disease or medication/not any bone-affecting disease or medication) was formed according to self-reported morbidities and medications in the 5-year postal inquiry. The diseases in this one dichotomous variable included renal disease, liver disease, insulin-dependent diabetes, malignancies, rheumatoid arthritis, endocrine abnormalities (parathyroid/thyroid glands, adrenals), malabsorption (including lactose malabsorption), total/partial gastrectomy, postovariectomy status, premenopausal amenorrhea, alcoholism, and long-term immobilization. The medication included: corticosteroids, thyroid medication, diuretics, cytotoxic drugs, anticonvulsive drugs, anabolic steroids, calcitonin, bisphosphonates, and vitamin D. The percentage of women with bone-affecting diseases did not differ significantly between the present study population (*n* = 1652) and total OSTPRE population sample (37% versus 45%, resp., (*P* > .100)).

## 3. Results

### 3.1. Characteristics of the Study Population

Table 1 describes the baseline and follow-up characteristics according to the mean annual bone loss rate tertiles. The tertiles of bone loss rate were (1) high bone loss rate: over 0.84% per year, (2) moderate bone loss rate: 0.13% to 0.84% per year, and (3) low bone loss rate: under 0.13% per year. Women with high bone loss rate had significantly less weight gain during the baseline to 5-year measurement, less HT use, and more bone-affecting medications or diseases. Women with high or moderate bone loss had lower grip strength compared to women with low bone loss. In addition, the fracture types according to study groups are presented in Table 1. The statistically significant differences in the characteristics were taken into account by including them in multivariate analyses of the present study.

### 3.2. Risk Factors for Fractures According to Bone Loss Rate

**Figure 2:**
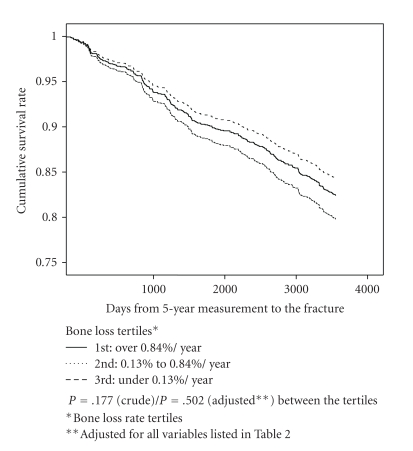
Cumulative 15-year fracture-free survival rate for any fragility fracture according to annual bone loss rate (tertiles)*. Cox proportional hazard model (*n* = 1652).

Figure 2 represents the absolute fracture-free survival according to bone loss tertiles in Cox proportional hazards model. Accordingly, there were no differences in fracture-free survival between the tertiles (*P* = .177 (univariate)/*P* = .502 (adjusted) between high and low annual bone loss rate tertiles). 

Table 2 represents the risk factors for follow-up fractures according to bone loss rate tertiles in uni- and multi-variate Cox proportional hazards model. Among women with high bone loss rate (over 0.84% per year) high T-score (*P* = .012, HR = 0.707; 95% confidence interval 0.539–0.927), no sister's fracture (*P* = .001, HR = 0.346; 0.187–0.641) and no mother's fracture (*P* = .015, HR = 0.518; 0.305–0.878) were associated with lower risk of fractures in both uni- and multi-variable models. 

 In women with moderate bone loss rate (0.13–0.84% per year), high T-score (*P* = .008, HR = 0.692; 0.527–0.908) and good grip strength (*P* = .022, HR = 0.982; 0.967–0.997) were the only significant predictors of higher fracture-free survival rate in multi-variate model. In univariate model, no use of HT in postmenopause was additional significant predictor of higher fracture risk among these women (*P* < .001). 

 In women with low bone loss rate (under 0.13% per year) there were no significant predictors of fracture risk observed among the variables investigated in multivariate model. In univariate model high grip strength was the only significant predictor of higher fracture-free survival (OR = 0.986, 95% confidence interval 0.974–0.999, *P* = .032). 

**Figure 3:**
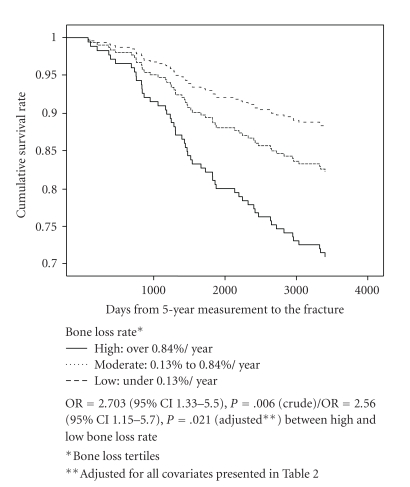
Effect of bone loss rate on fracture risk among women with self-reported mother's fracture. Cox proportional hazards model (*n* = 319).

 For the significant predictors presented in Table 2, a statistically significant interaction was confirmed for sister's fracture (*P* < .001) and mother's fracture (*P* = .020) in Cox proportional hazards model. Based on these results, the effect of bone loss on fracture risk was further investigated among women with mother's and sister's fracture. In women with sister's fracture, high bone loss rate (over 0.84% per year) predicted lower fracture-free survival rate in comparison to low bone loss rate (*P* = .039, OR = 2.656, 95% confidence interval 1.052–6.703) in multivariate model but not in univariate model (*P* = .410).  Figure 3 represents the effect of bone loss rate on fracture-free survival according to mother's fracture. Accordingly, high bone loss rate predicted lower fracture-free survival rate in comparison to low bone loss rate in univariate (OR = 2.703; 1.33–5.50, *P* = .006) and adjusted multivariate (OR = 2.560; 1.15–5.70, *P* = .021) models. For women with no sister's or mother's fracture these effects were not seen.

## 4. Discussion

The present study investigated the risk factors for early postmenopausal fractures according to bone loss rate, in a study cohort of 1652 Finnish women. Bone loss rate itself was not a significant predictor of fractures in total population. However, the risk factors for low-trauma energy fractures were found to differ according to rate of bone loss. In women with high bone loss rate, sister's and mother's fractures were strongly related to increased fracture risk, in addition to negative effects of low baseline BMD. In women with low bone loss rate, there were no significant predictors found for fracture risk in multivariate model. A statistically significant interaction with bone loss rate was observed for sister's and mother's fracture. Accordingly, high bone loss rate was a predictor of higher fracture risk among women with sister's or mother's fracture.

The strengths of the present study included its prospective and population-based nature, large base population, and long-term follow-up interval. Also, the present study setting can be considered optimal for investigating the hypothesis for present study. The BMD follow-up period was similar between the study-groups, and bone mass and grip strength measurements, as well as anthropometric measurements, were performed under the supervision of trained personnel. This may suppress occasional confounders due to measurement errors. All the self-reported follow-up fractures were validated from the medical records by study group physicians. Finally, adjustment for bone-affecting diseases and medications with one combined dichotomous variable (any bone-affecting disease or medication/not any bone affecting disease or medication) was used in the multivariate models, which may have weakened the possible bias caused by varying sampling fractions. While the use of dichotomous variable was not ideal for investigating the effects of individual morbidities and medications, for adjustment purposes it provided a practical choice in order to reduce the number of covariates into minimum in multivariate models.

A possibility of uncontrolled confounding is always present in epidemiological studies. Although the study sample of 2025 women was randomly selected from the base population of 14 220 women; the final study sample of 1652 women presented a relatively small part of the original OSTPRE cohort. Accordingly, this heavy selection process includes the risk that the final sample may not be fully representative of the underlying population. The selection procedure may additionally cause lack of power. The percentage (37%) of bone-affecting conditions was high due to the wide spectrum of diseases or medications considered to potentially have such properties. This variable was, however, not associated with fracture risk in any of the present analyses. Considering the predictive variables in the present analyses, we used mean not maximum grip strength which, however, may be equally consistent [30]. In addition, the definition of menopausal transition in the present study was based purely on self-reports according to amenorrhea, without information on hormonal levels. Self-reports, however, have proved to be quite accurate in this matter [31]. There were some differences in characteristics between the study groups. As an example, women with high bone loss rate had less weight gain and HT use. These differences between the bone loss groups were taken into account by adjustments in the multivariate models.

The present study used the baseline and 5-year BMD measurements for defining bone loss rate and it was used to predict fractures between 5- to 15-year follow-up period. In addition, 5-year postal inquiry information was used as additional predictive data for fractures. The reason for the use of only the first two DXA measurements was the aim to investigate the causal relationship between bone loss and fractures. Accordingly, the possible changes in bone loss rate during the fracture follow-up may have occurred.

Previously, several studies have found that first-degree relatives' fractures, especially those of mother's, are associated with the postmenopausal incidence of fractures [13–18]. In our previous report we showed that sister's fracture history is, in general, associated with perimenopausal fractures [32]. The present study confirms that sister's fracture history is a relevant determinant of perimenopausal fractures among Finnish women but adds to this finding that bone loss may interact with this genetic tendency for bone fragility. In addition, the present study found that mother's fractures were negatively associated with fracture-free survival only among women with high bone loss rate. It should be remembered, however, that questions concerning the parents fracture history may provide less valid answers which may contribute to the lack of association with fracture incidence in other study groups. The background of the genetics of fragility fractures has been reviewed recently [33]. Also, sister's and mother's fractures showed significant interaction with bone loss rate in Cox model in the present study, and especially among women with mother's fracture, bone loss was predictive for future fractures. This finding suggests that among postmenopausal women with first-degree female relatives fractures bone loss may be used in evaluation of fracture risk. In the present study, we also included the relatives' fractures during the follow-up, in addition to previous fractures, for two reasons. Firstly, the history of sister's and mother's wrist and hip fractures was not questioned earlier than 10-year follow-up postal inquiry in the present study. Secondly, it is unlikely that genetic predisposition to fractures would be determined during the present follow-up interval, thus eliminating the problem with regards causality.

Our results indicate that bone loss rate as such is not associated with increased fracture risk if abovementioned risk fractures was not present. However, bone loss rate may alter the effects of other risk factors related to increased fracture risk. In the present study the effects of some life-style and anthropometric risk factors on fractures were dependent on bone loss rate. As an example, good grip strength seemed to prevent fractures in women with moderate bone loss rate. Grip strength has been previously shown to reflect overall physical performance [34] and to be associated with perimenopausal bone loss and incidence of fractures [26, 35]. Similarly, T-score was strongly predictive for fractures in groups with moderate to high bone loss rate, but not among women with low bone loss rate. Of these previously discussed variables, a statistically significant interaction was, however, not confirmed in Cox interaction model.

The use of hormone therapy should not be considered primary medication for osteoporosis because of possible serious adverse events [36] and also due to the introduction of newer effective nonhormonal bone medications. However, there still may be indications for HT in other than bone related conditions for perimenopausal women. In the present study, we aimed primarily at using HT as a confounder of menopause status rather than as a preventive medication for fractures or low bone mass. In the present study HT was protective against fragility fractures only in women with moderate bone loss in univariate model. In multivariate model this effect was not seen which suggests that other factors may outweigh HTs effects. The bone protective effects and biology of HT have been studied in detail previously [37, 38].

The purpose of the present study was to demonstrate that risk factors for fractures differ according to bone loss rate. The current diagnosis and definition of osteoporosis is based on BMD measurements, although it has been speculated that bone quality, including several other aspects of bone biology apart from BMD, is a more appropriate measure. As an example, it has been stated that only 44% of nonvertebral fractures occur among osteoporotic women while the majority of these fractures occur in women with higher BMD [39]. This fact underlines the need to evaluate fracture risk more holistically than BMD alone and suggests that there are several other properties of bone involved in high fragility [40, 41]. However, the interaction of risk factors for fragility fractures with bone loss rate, observed in the present study, may indicate that bone loss rate reflects additional properties of bone health besides single BMD measurement. To our knowledge, this is the first population-based long-term follow-up study to show that risk factors for fragility fractures are dependent on rate of bone loss in postmenopausal women with especially strong interaction with first-degree female relative's fractures.

In conclusion, the risk factors for fragility fractures seem to interact with bone loss rate among perimenopausal women. In women with high bone loss rate, there may be especially strong genetic predisposition to increased bone fragility. Also, in women with low bone loss rate, BMD seems not to predict fractures in contrast to women with moderate to high bone loss rate. It may be concluded that while bone mass measurements do not fully explain bone fragility among elderly women, it may reveal differences in response to other fracture risk factors. However, bone loss rate, as such, may not be considered predictive for fractures without contribution of other concomitant risk factors, such as first-degree female relatives fractures. These results should be confirmed in other populations and also among elderly males.

## Figures and Tables

**Table 1:** Characteristics according to study groups (annual bone loss tertiles, *n* = 1652).

Characteristics				
	Bone loss tertiles*			
	High (*n* = 551)	Moderate (*n* = 547)	Low (*n* = 553)	Total (*n* = 1652)
(A) Means (SD) of continuous variables				
Years since menopause (at 5 years)	7.7(5.1)	8.0(5.5)	8.3(6.1)	8.0(5.6)
Baseline age, years	53.5(2.8)	53.6(3.01)	53.6(2.9)	53.5(2.9)
Baseline height cm	161.4(5.3)	161.4(5.1)	161.1(5.0)	161.3(5.2)
Baseline weight kg	69.6(12.3)	69.1(11.6)	68.4(10.7)	69.0(11.6)
Weight change (Bl to 5 years), kg	2.1(5.3)	2.7(4.9)	4.2(5.1)	3.0(5.2)^††^
Grip strength kPA	62.6(16.0)	60.8(16.6)	64.7(16.4)	62.7(16.4)^††^
Calcium intake mg/day	797(294)	779(290)	809(284)	795(290)
Alcohol intake g/week	39.0(75.2)	48.2(76.3)	49.3(73.0)	45.52(75.0)^†^
Baseline FN T-score SD	−0.30(1.05)	−0.35(1.07)	−0.54(1.09)	−0.40(1.07)^††^
5-year FN T-score SD	−0.99(0.99)	−0.58(1.04)	−0.33(1.12)	−0.63(1.08)^††^
(B) Distribution of category variables (%)				
Menopause status (at 5 years)				
Premenopuasal (no HT)	13.1	8.2	7.8	9.7
Postmenopausal (no HT)	35.4	30.2	23.9	29.8
HT use	51.5	61.6	68.4	60.5^††^
No bone-affecting disease/medication**	53.9	48.4	45.2	49.2^†^
Mother's fracture	18.1	19.5	20.3	19.3
Sister's fracture	9.6	10.3	11.4	10.5
Any previous fracture at 5 years	20.3	20.5	19.2	20.0
Smoking (never/ever)	19.2	17.4	18.4	18.4
Decline in grip strength (Bl to 5years)	28.3	22.9	24.3	25.2
Follow-up fractures	25.2	28.3	24.2	25.9
Wrist	9.4	9.1	8.5	9.0
Unimalleolar ankle	4.0	3.7	4.0	3.9
Other ankles	1.3	1.1	0.7	1.0
Lumbar/thoracal spine	2.4	2.0	1.3	1.9
Humerus	1.3	0.9	0.4	0.8

*High: over 0.84% per year, moderate: 0.13% to 0.84% per year, low: under 0.13% per year. ^†^ *P* < .05/ ^††^ *P* < 0.01 in ANOVA (continuous variables) and Chi square test (categorical variables) between the study groups. **For specification of the diseases and medications included, see Materials and Methods—Statistical Methods.

**Table 2:** Predictors of 15-year fracture free-survival according to bone loss rate tertiles. Uni- and multi-variate Cox proportional hazards models* (*n* = 1652).

Variables	Univariate Sig.	HR (95% CI)	Multivariate Sig.	HR (95% CI)
High bone loss rate				
Grip strength	0.294	0.993(0.981–1.006)	0.290	0.992(0.977–1.007)
Grip strength change	0.200	1.501(0.807–2.791)	0.609	1.200(0.596–2.416)
No previous fracture	0.052	0.584(0.339–1.004)	0.472	0.785(0.406–1.518)
Age	0.348	0.965(0.895–1.040)	0.115	0.929(0.847–1.018)
Body mass index	0.174	0.964(0.914–1.016)	0.348	0.968(0.906–1.036)
Weight change	0.727	0.992(0.951–1.036)	0.433	0.980(0.932–1.031)
Use of HT**	0.866		0.919	
Premenopausal (no HT)	0.748	1.106(0.598–2.047)	0.713	0.864(0.397–1.880)
Postmenopausal (no HT)	0.750	0.926(0.579–1.482)	0.969	1.012(0.559–1.832)
Bone-affecting condition^†^	0.837	0.957(0.628–1.458)	0.917	1.030(0.594–1.785)
Nutritional calcium intake	0.859	1.000(0.999–1.001)	0.993	1.000(0.999–1.001)
Alcohol intake	0.808	1.000(0.997–1.003)	0.367	0.998(0.994–1.002)
Smoking	0.595	1.253(0.547–2.870)	0.619	1.266(0.500–3.201)
No sister's fracture	0.000	0.358(0.204–0.630)	0.001	0.346(0.187–0.641)
No mother's fracture	0.004	0.506(0.318–0.806)	0.015	0.518(0.305–0.878)
Bone mineral density	0.007	0.746(0.602–0.925)	0.012	0.707(0.539–0.927)
Moderate bone loss rate				
Grip strength	0.003	0.983(0.972–0.994)	0.022	0.982(0.967–0.997)
Grip strength change	0.545	1.169(0.705–1.940)	0.667	0.881(0.494–1.572)
No previous fracture	0.091	0.630(0.369–1.076)	0.239	0.686(0.366–1.285)
Age	0.004	1.097(1.030–1.168)	0.516	1.027(0.947–1.115)
Body mass index	0.191	0.968(0.921–1.016)	0.782	0.991(0.929–1.057)
Weight change	0.721	0.993(0.953–1.034).	0.742	1.008(0.959–1.060)
Use of HT**	0.000		0.104	
Premenopausal (no HT)	0.154	0.428(0.134–1.373)	0.315	0.536(0.159–1.808)
Postmenopausal (no HT)	0.001	2.017(1.354–3.005)	0.104	1.579(0.910–2.742)
Bone-affecting condition^†^	0.111	1.378(0.929–2.044)	0.416	1.249(0.731–2.133)
Nutritional calcium intake	0.752	1.000(0.999–1.000)	0.851	1.000(0.999–1.001)
Alcohol intake	0.843	1.000(0.997–1.002)	0.624	1.001(0.998–1.003)
Smoking	0.297	1.702(0.626–4.628)	0.583	1.341(0.470–3.825)
No sister's fracture	0.055	0.563(0.313–1.014)	0.106	0.594(0.316–1.116)
No mother's fracture	0.478	1.208(0.716–2.038)	0.460	1.258(0.684–2.313)
Bone mineral density	0.000	0.632(0.521–0.768)	0.008	0.692(0.527–0.908)
Low bone loss rate				
Grip strength	0.032	0.986(0.974–0.999)	0.155	0.987(0.970–1.005)
Grip strength change	0.207	0.702(0.405–1.216)	0.147	0.621(0.327–1.182)
No previous fracture	0.064	0.559(0.302–1.035)	0.070	0.518(0.254–1.056)
Age	0.309	1.041(0.964–1.123)	0.904	1.006(0.917–1.102)
Body mass index	0.239	1.031(0.980–1.085)	0.778	1.011(0.935–1.095)
Weight change	0.166	1.029(0.988–1.071)	0.203	1.032(0.983–1.084)
Use of HT**	0.401		0.770	
Premenopausal (no HT)	0.628	0.778(0.281–2.152)	0.984	0.988(0.308–3.173)
Postmenoapusal (no HT)	0.240	1.350(0.819–2.225)	0.504	1.267(0.634–2.531)
Bone-affecting condition^†^	0.816	0.948(0.604–1.489)	0.791	0.918(0.487–1.730)
Nutritional calcium intake	0.053	0.999(0.999–1.000)	0.174	0.999(0.999–1.000)
Alcohol intake	0.758	0.999(0.996–1.003)	0.544	1.001(0.997–1.005)
Smoking	0.299	1.705(0.623–4.666)	0.294	1.904(0.572–6.335)
No sister's fracture	0.065	0.544(0.285–1.039)	0.259	0.651(0.309–1.371)
No mother's fracture	0.269	1.434(0.756–2.719)	0.710	1.142(0.567–2.299)
Bone mineral density	0.060	0.818(0.663–1.009)	0.105	0.785(0.585–1.052)

*Bone loss tertiles: high: over 0.84% per year, moderate: 0.13% to 0.84% per year, low: under 0.13% per year. ^†^Bone-affecting disease or medication, dichotomous variable (any disease or medication/not any disease or medication). See materials and Methods for details. **Reference group.
